# A Comparative Analysis of the Hinotori and da Vinci Robotic Systems in Total Hysterectomy for Benign Uterine Gynecological Diseases Using Propensity Score Matching

**DOI:** 10.1111/ases.70327

**Published:** 2026-06-14

**Authors:** Yutaka Torii, Chiaki Oshima, Ryoma Aoki, Akira Yasue, Kazuhiko Tsukada, Haruki Nishizawa

**Affiliations:** ^1^ Department of Obstetrics and Gynecology, School of Medicine Fujita Health University Toyoake Aichi Japan; ^2^ Department of Gynecology Fujita Health University Okazaki Medical Center Okazaki Aichi Japan

**Keywords:** propensity score, robotic surgical procedures, uterine diseases

## Abstract

**Introduction:**

The hinotori surgical robot system, the first Japanese‐made robotic‐assisted surgical system to receive Japanese regulatory approval in 2020, is now being applied clinically in gynecology. This study aimed to comprehensively compare the hinotori and the da Vinci Xi Surgical System with respect to safety and surgical outcomes in robot‐assisted total hysterectomy for benign uterine gynecological diseases.

**Methods:**

This retrospective observational study used 1:1 propensity score matching (PSM) to review patients who underwent robot‐assisted total hysterectomy for benign uterine gynecological diseases at our hospital from August 2020 to June 2025. The hinotori was used in 40 patients, while the da Vinci was used for 187 patients. The primary endpoint was the incidence of complications of Grades I–V according to the Clavien–Dindo (CD) classification system within 30 days postoperatively.

**Results:**

After PSM, 40 patients from each group were included in the analysis. Patient characteristics were well balanced between the groups. Complication rates for CD Grades I–V were 7.5% in the hinotori group and 10.0% in the da Vinci group, with no significant difference (*p* = 0.602). Meanwhile, operative time (144 vs. 118 min, *p* < 0.001) and console time (113 vs. 76 min, *p* < 0.001) were significantly longer in the hinotori group.

**Conclusion:**

In robot‐assisted total hysterectomy for benign uterine gynecological diseases, the hinotori and da Vinci were comparable in terms of complication incidence. However, longer operative and console times were observed with hinotori, possibly reflecting early adoption effects or platform‐specific factors.

## 1 Introduction

Minimally invasive surgery in Japan is rapidly shifting from laparoscopic to robot‐assisted techniques. Robot‐assisted surgery is becoming popular worldwide as a standard technique for minimally invasive surgery because it offers several advantages, such as three‐dimensional high‐resolution stereoscopic visualization, an anti‐vibration mechanism, and excellent maneuverability with multi‐joint robotic arms that provide a high degree of freedom and precise movement [1, 2, 3]. Several studies in gynecology have reported that robot‐assisted surgery reduces blood loss, hospital stay, and postoperative complications in total hysterectomy for benign uterine diseases compared with open or laparoscopic surgery [4, 5, 6, 7]. The da Vinci Surgical System (Intuitive Surgical Inc., U.S.A.; hereinafter “da Vinci”) was approved by the U.S. Food and Drug Administration in July 2000. In Japan, the Ministry of Health, Labour, and Welfare's Pharmaceutical Affairs and Food Sanitation Council approved da Vinci for manufacturing and distribution in 2009. It later received insurance coverage for urological procedures in April 2012 and for total hysterectomy in gynecology in 2018. Although robot‐assisted surgery using the da Vinci system was introduced and its indications expanded, the hinotori received manufacturing and marketing approval as Japan's first surgical robot system for urology surgery in August 2020 [8], followed by the expansion of insurance coverage to include gastroenterological surgery and gynecology in December 2022.

The hinotori Surgical Robot System (Medicaroid Corporation, Japan; hereinafter, “hinotori”) was developed by integrating the robotics technology of Kawasaki Heavy Industries, a leading industrial robotics company, with the medical expertise of Sysmex Corporation. In urology, where insurance coverage was first implemented, studies have reported the safety and feasibility of hinotori in total prostatectomy and partial nephrectomy [9, 10], as well as comparisons with the da Vinci system [11, 12, 13]. In gynecology, Togami et al. reported the use of hinotori in robot‐assisted total hysterectomy (robot‐assisted simple hysterectomy, RSH) in 2023, demonstrating its safety and feasibility [14]. Since then, some reports have compared the hinotori system with the preceding da Vinci system in RSH and reported comparable surgical outcomes; however, there is a paucity of data on a comprehensive comparison between the two systems, particularly regarding detailed comparisons of surgical efficiency and safety [15, 16, 17, 18].

This study aimed to retrospectively compare the hinotori and da Vinci systems with respect to perioperative outcomes in RSH for benign uterine diseases using propensity score matching (PSM), and to evaluate the safety and effectiveness of the hinotori system during the early postinduction period.

## 2 Materials and Methods

### 2.1 Target Patients and Study Design

**FIGURE 1:**
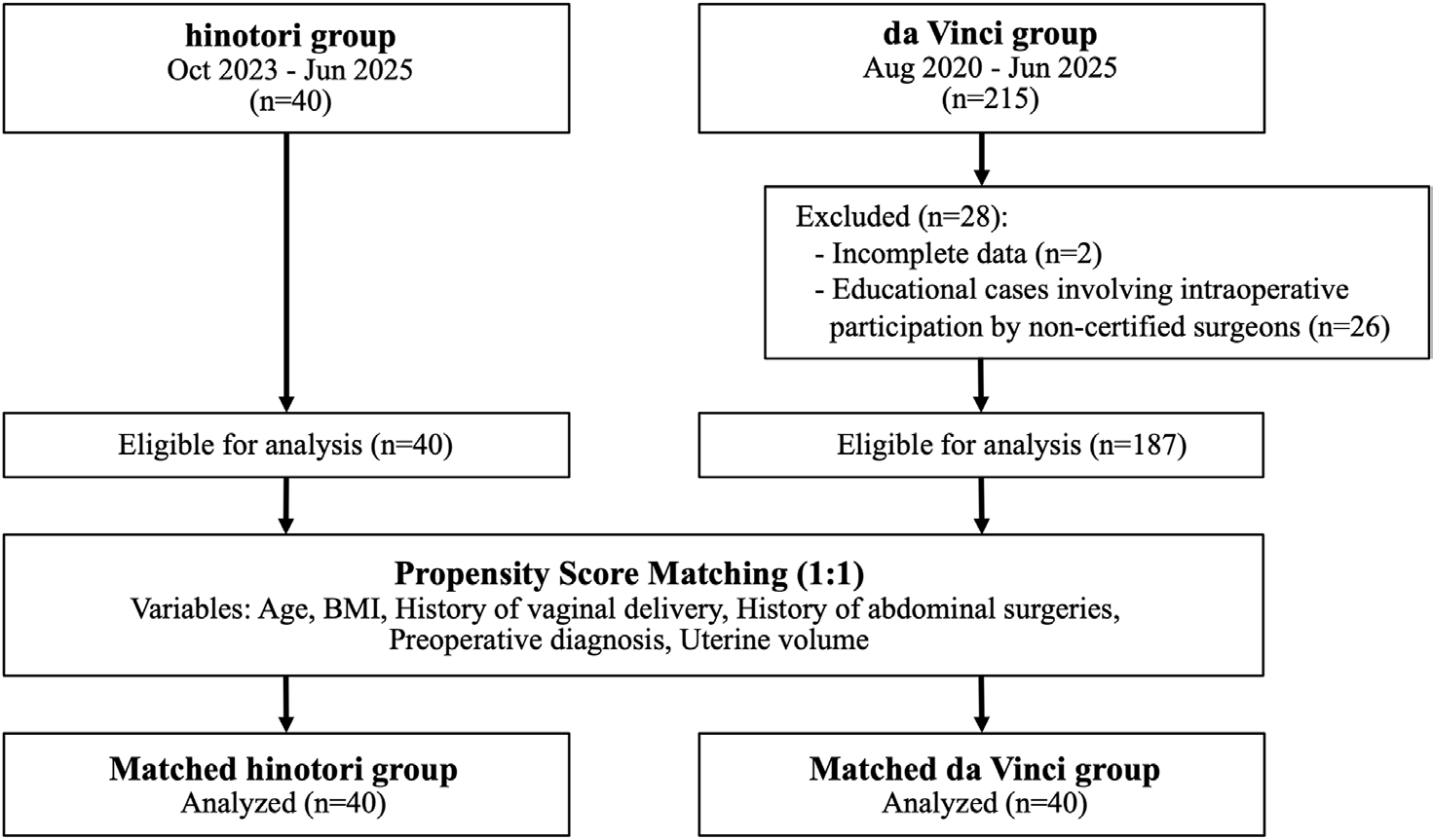
Flowchart of patient selection and propensity score matching (PSM). After exclusion of 2 cases with incomplete data and 26 educational cases from the da Vinci cohort, 187 da Vinci cases, and 40 hinotori cases were eligible for analysis. After 1:1 PSM, 40 patients from each group were included in the matched cohort.

This was a single‐center, retrospective observational study. Consecutive cases involving patients preoperatively diagnosed with benign uterine diseases (uterine myoma, uterine adenomyosis, endometrial polyp, endometrial hyperplasia, and cervical intraepithelial dysplasia) who underwent RSH in the gynecology department of our hospital between August 2020 and June 2025 were retrospectively reviewed. The hinotori group consisted of 40 cases treated from October 2023 to June 2025. The da Vinci group initially comprised 215 cases in which RSH was performed using the da Vinci Xi system between August 2020 and June 2025. Following the exclusion of 2 cases with incomplete data and 26 educational cases, 187 cases were included in the final analysis (Figure 1). Educational cases were defined as cases in which a surgeon without certification in laparoscopic or robotic surgery by the Japan Society of Gynecologic and Obstetric Endoscopy (JSGOE) performed any part of the intraoperative surgical procedure. The same exclusion criterion was applied to the hinotori cohort; however, no hinotori cases met this definition. All surgical procedures in the final analytic cohort were conducted by JSGOE‐certified surgeons in laparoscopic or robotic surgery.

PSM was performed to reduce baseline differences in preoperative patient characteristics between the hinotori and da Vinci groups and to assess whether the observed perioperative findings were consistent after adjustment for observable confounders. Age, body mass index (BMI), history of vaginal delivery, history of abdominal surgeries, preoperative diagnosis, and uterine volume (calculated as 4/3π multiplied by half the uterine longitudinal, anteroposterior, and transverse diameters) served as matching variables. These variables were selected because they were preoperative factors considered clinically relevant to surgical difficulty and perioperative outcomes. Preoperative diagnosis was categorized as myoma/adenomyosis or other benign indications to avoid sparse diagnostic categories and improve the stability and interpretability of the propensity score model. Two da Vinci cases with incomplete data were excluded before PSM, and no imputation was performed. Propensity scores were then calculated using logistic regression models. A 1:1 nearest‐neighbor matching approach was implemented with a caliper width of 0.2 standard deviation of the propensity scores. Covariate balance following matching was assessed using standardized mean differences. A standardized mean difference below 0.1 was interpreted as evidence of adequate balance for the variable.

The primary endpoint was the incidence of complications of Grades I–V according to the CD classification within 30 days postoperatively. Secondary endpoints included measures of surgical efficiency (operative time, interval from incision to console activation, and console time), safety (blood loss and conversion rate), and postoperative outcomes (length of postoperative hospital stay, white blood cell counts, and C‐reactive protein (CRP) levels on postoperative Days 1 and 3, as well as the rates of prolonged hospital stay and readmission within 30 days postoperatively).

### 2.2 Robotic Systems and Surgical Procedure

RSH was performed using either the hinotori Surgical Robot System or the da Vinci Xi system according to our institutional standardized procedure. The hinotori system has four robotic arms and a docking‐free design with software‐based pivot calibration [19]. In both groups, all procedures were performed using a four‐port technique without an assistant port. The standardized surgical steps included bladder dissection, visual confirmation of ureteral peristalsis, parametrial dissection, uterine artery coagulation and cutting, circumferential colpotomy, transvaginal uterine extraction, and double‐layer vaginal cuff closure.

### 2.3 Statistical Analysis

All statistical analyses were conducted using EZR version 1.68 (https://www.jichi.ac.jp/saitama‐sct/SaitamaHP.files/download.html) [20]. EZR is a statistical software package that extends the capabilities of both R and R Commander. Continuous variables were compared using the Mann–Whitney *U* test, and categorical variables were compared using Fisher's exact test. These tests were applied to group comparisons both before and after PSM. After matching, they were used as descriptive group‐level comparisons within the matched cohort, and post matching *p*‐values were interpreted together with standardized mean differences and the clinical magnitude of observed difference. A two‐tailed *p*‐value of < 0.05 was considered statistically significant.

To examine whether operative time changed with institutional experience, operative time was plotted against the sequential case number within each platform. Locally weighted scatterplot smoothing curves were overlaid to visualize temporal trends. Console time was additionally plotted in the supplementary learning‐curve analysis.

### 2.4 Research Ethics

The Medical Research Ethics Review Committee of the School of Medicine at Fujita Health University (Approval No. HM23‐251) approved this comprehensive research study, which was conducted in accordance with the principles of the Declaration of Helsinki. Information regarding this research was provided to study participants through the “Disclosure of Information on Medical Research Involving Human Subjects” section of the Fujita Health University website (https://fujita.bvits.com/esct/publish), and they were allowed to indicate their willingness not to consent to the study in an opt‐out format.

## 3 Results

### 3.1 Study Population

**FIGURE 2:**
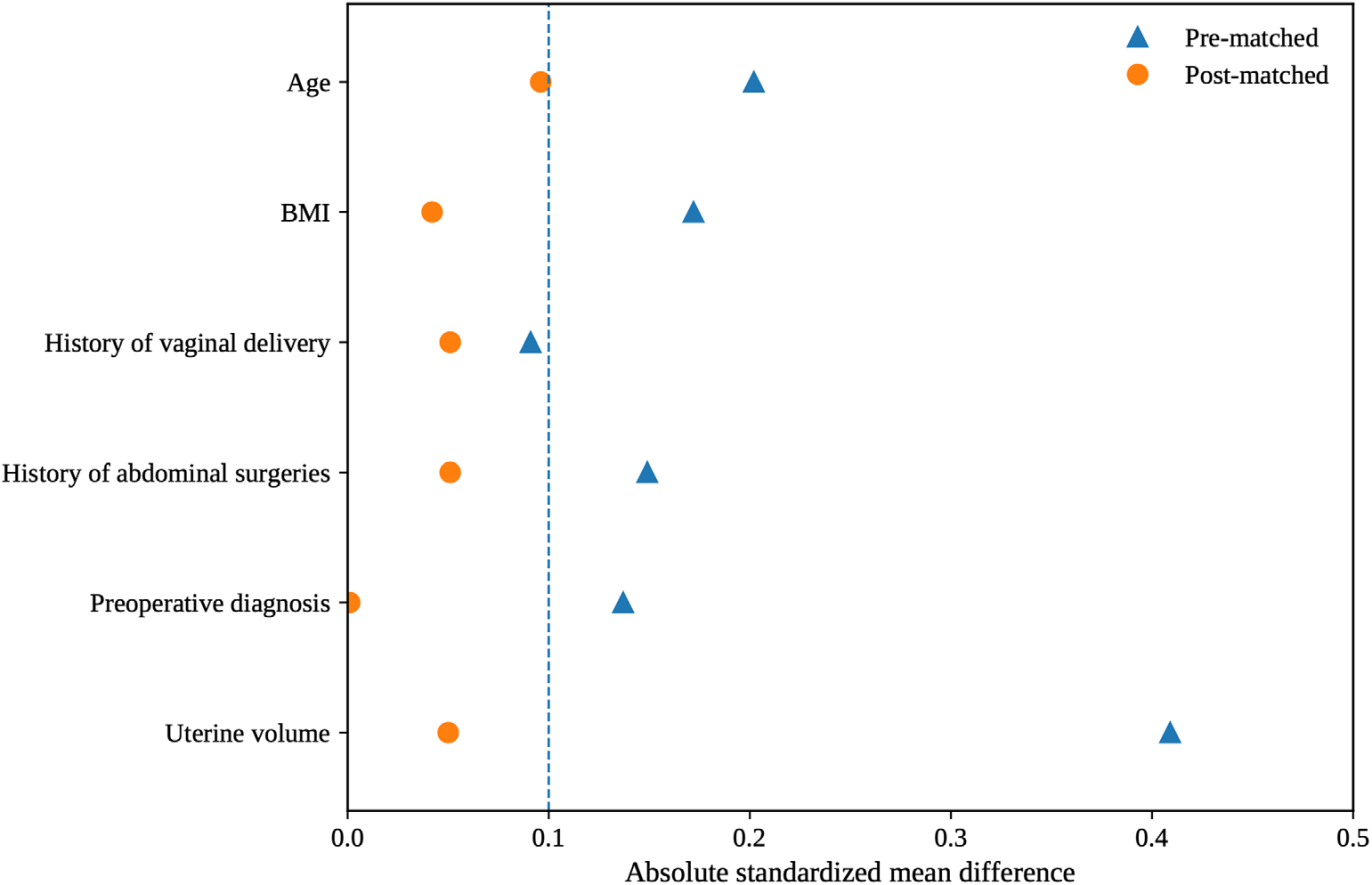
Love plot showing covariate balance before and after propensity score matching. The plot shows absolute standardized mean differences for covariates included in the propensity score model; blue triangles indicate values before matching, and orange circles indicate values after matching. The dashed vertical line indicates the 0.1 threshold for adequate balance.

Table 1 presents the patient characteristics before and after PSM. After 1:1 PSM according to the patient selection flow shown in Figure 1, 40 cases from each group were included in the matched cohort. There were no significant differences between the two groups in age, BMI, history of vaginal delivery, history of abdominal surgeries, or preoperative diagnosis before or after PSM. Before PSM, the da Vinci group had a significantly larger uterine volume. After PSM, patient characteristics were well balanced between the two groups, with all absolute standardized mean differences below 0.1. Covariate balance before and after matching is also shown in the Love plot (Figure 2).

**TABLE 1:** Patient characteristics.

Characteristic	Pre‐matched			SMD	Post‐matched			SMD
	hinotori (*n* = 40)	da Vinci Xi (*n* = 187)	*p*		hinotori (*n* = 40)	da Vinci Xi (*n* = 40)	*p*	
Age, years	47 [42.8–49.3]	47 [44–50]	0.28	0.202	47 [42.8–49.3]	47 [43–50]	0.668	0.096
BMI, kg/m^2^	21.9 [19.6–25.9]	22.4 [20.2–24.5]	0.279	0.172	21.9 [19.6–25.9]	22.3 [20.8–25.9]	0.851	0.042
History of vaginal delivery			0.587	0.091			1	0.051
No	15 (37.5%)	62 (33.2%)			15 (37.5%)	16 (40%)		
Yes	25 (62.5%)	125 (66.8%)			25 (62.5%)	24 (60%)		
History of abdominal surgeries			0.454	0.149			1	0.051
0	25 (62.5%)	130 (69.5%)			25 (62.5%)	24 (60%)		
≥ 1	15 (37.5%)	57 (30.5%)			15 (37.5%)	16 (40%)		
Preoperative diagnosis			0.415	0.137			1	< 0.001
Uterine myoma, uterine adenomyosis	37 (92.5%)	179 (95.7%)			37 (92.5%)	37 (92.5%)		
Others (endometrial polyp, endometrial hyperplasia, CIN)	3 (7.5%)	8 (4.3%)			3 (7.5%)	3 (7.5%)		
Uterine volume, cm^3^	227.9 [121.4–330.8]	259.8 [154.3–486]	0.03	0.409	227.9 [121.4–330.8]	207.8 [120.9–296.2]	0.823	0.05

*Note:* Values are presented as median [interquartile range] or number (%).

Abbreviation: SMD, standardized mean difference.

### 3.2 Surgical Outcomes

Table 2 presents the surgical outcomes before and after PSM. Console time refers to the duration of robotic console operation and was calculated as operative time minus setup time and the time from robotic system roll‐out to completion of surgery. Actual console time refers to console time excluding the duration required for transvaginal extraction of the detached uterus. Setup time refers to the duration from skin incision to completion of instrument placement, when console surgery could be initiated. Roll‐in time, which was included in setup time, refers to the duration from skin incision to completion of robotic system roll‐in after trocar placement and establishment of the pelvic operative field in the Trendelenburg position.

**TABLE 2:** Surgical outcomes.

Outcome	Pre‐matched			Post‐matched		
	hinotori (*n* = 40)	da Vinci Xi (*n* = 187)	*p*	hinotori (*n* = 40)	da Vinci Xi (*n* = 40)	*p*
Operative time, min	144 [132.3–170.5]	130.5 [111.8–156]	0.003	144 [132.3–170.5]	118 [104–135]	< 0.001
Console time, min	113 [95.8–135]	85 [70–109.3]	< 0.001	113 [95.8–135]	76 [65.5–94.8]	< 0.001
Actual console time, min	107 [90–125]	78.5 [67–96.3]	< 0.001	107 [90–125]	72 [64.3–85.3]	< 0.001
Setup time, min	17 [13–23.8]	14 [11–18.3]	0.006	17 [13–23.8]	14 [10–18]	0.005
Roll‐in time, min	7 [5–11.8]	9 [7–13]	0.003	7 [5–11.8]	9 [6.3–12]	0.277
Blood loss, mL	10 [6–29.5]	30 [11.8–56]	< 0.001	10 [6–29.5]	20 [10–32.5]	0.163
Weight of the removed uterus, g	193 [112.5–273]	215.5 [127–368.5]	0.642	193 [112.5–273]	129 [105.3–249.8]	0.126
Conversion	0 (0%)	0 (0%)	1	0 (0%)	0 (0%)	1

Footnotes: Values are presented as median [interquartile range] or number (%).

After PSM, the hinotori group had significantly longer operative time (144 vs. 118 min, *p* < 0.001), console time (113 vs. 76 min, *p* < 0.001), actual console time (107 vs. 72 min, *p* < 0.001), and setup time (17 vs. 14 min, *p* = 0.005). There were no statistically significant differences in intraoperative blood loss (10 vs. 20 mL, *p* = 0.163) or in the weight of the removed uterus (193 vs. 129 g, *p* = 0.126) between the two groups. Additionally, no patients in either group required conversion to laparoscopic or open surgery.

### 3.3 Perioperative Course

Table 3 presents the perioperative course. Within 30 days postoperatively, perioperative complications of CD Grades I–V occurred in 3 cases (7.5%) in the hinotori group and 4 cases (10%) in the da Vinci group. There was no significant difference between the groups (*p* = 0.602). No complications of CD Grades IIIa–V were observed. Reported complications consisted of one case of repairable intraoperative intestinal serosal injury in the da Vinci group, while the remaining six cases involved vaginal cuff infection or pelvic peritonitis. White blood counts did not differ on postoperative Days 1 and 3. However, the CRP level on postoperative Day 1 was significantly higher in the hinotori group (*p* = 0.005). There were no significant differences between the two groups in the length of postoperative hospital stay (5 vs. 5 days, *p* = 0.674), the rate of prolonged postoperative hospital stay (7.5% vs. 0%, *p* = 0.241), or readmission within 30 days postoperatively (0% vs. 7.5%, *p* = 0.241).

**TABLE 3:** Postoperative course.

Outcome	Pre‐matched			Post‐matched		
	hinotori (*n* = 40)	da Vinci Xi (*n* = 187)	*p*	hinotori (*n* = 40)	da Vinci Xi (*n* = 40)	*p*
Clavien‐Dingo classification			0.721			0.602
0	37 (92.5%)	161 (86.1%)		37 (92.5%)	36 (90%)	
1	0 (0%)	4 (2.1%)		0 (0%)	1 (2.5%)	
2	3 (7.5%)	22 (11.8%)		3 (7.5%)	3 (7.5%)	
WBC, /μL						
POD1	9250 [8100–10 825]	9500 [8075–10 925]	0.904	9250 [8100–10 825]	9450 [7725–10 650]	0.787
POD3	5500 [4800–6325]	5400 [4500–6425]	0.642	5500 [4800–6325]	5300 [4325–6650]	0.509
CRP, mg/dL						
POD1	1 [0.8–2.61]	1.01 [0.59–1.74]	0.009	1 [0.8–2.61]	1 [0.53–1.56]	0.005
POD3	1 [0.57–2.95]	1.41 [0.6–2.75]	0.961	1 [0.57–2.95]	1 [0.41–1.55]	0.119
Length of postoperative hospital stay, days	5 [5–5]	5 [5–5]	0.475	5 [5–5]	5 [5–5]	0.674
Prolonged postoperative hospital stay	3 (7.5%)	8 (4.3%)	0.415	3 (7.5%)	0 (0%)	0.241
Readmission within 30 days postoperatively	0 (0%)	9 (4.8%)	0.367	0 (0%)	3 (7.5%)	0.241

*Note:* Values are presented as median [interquartile range] or number (%).

### 3.4 Temporal Trends in Operative Time

**FIGURE 3:**
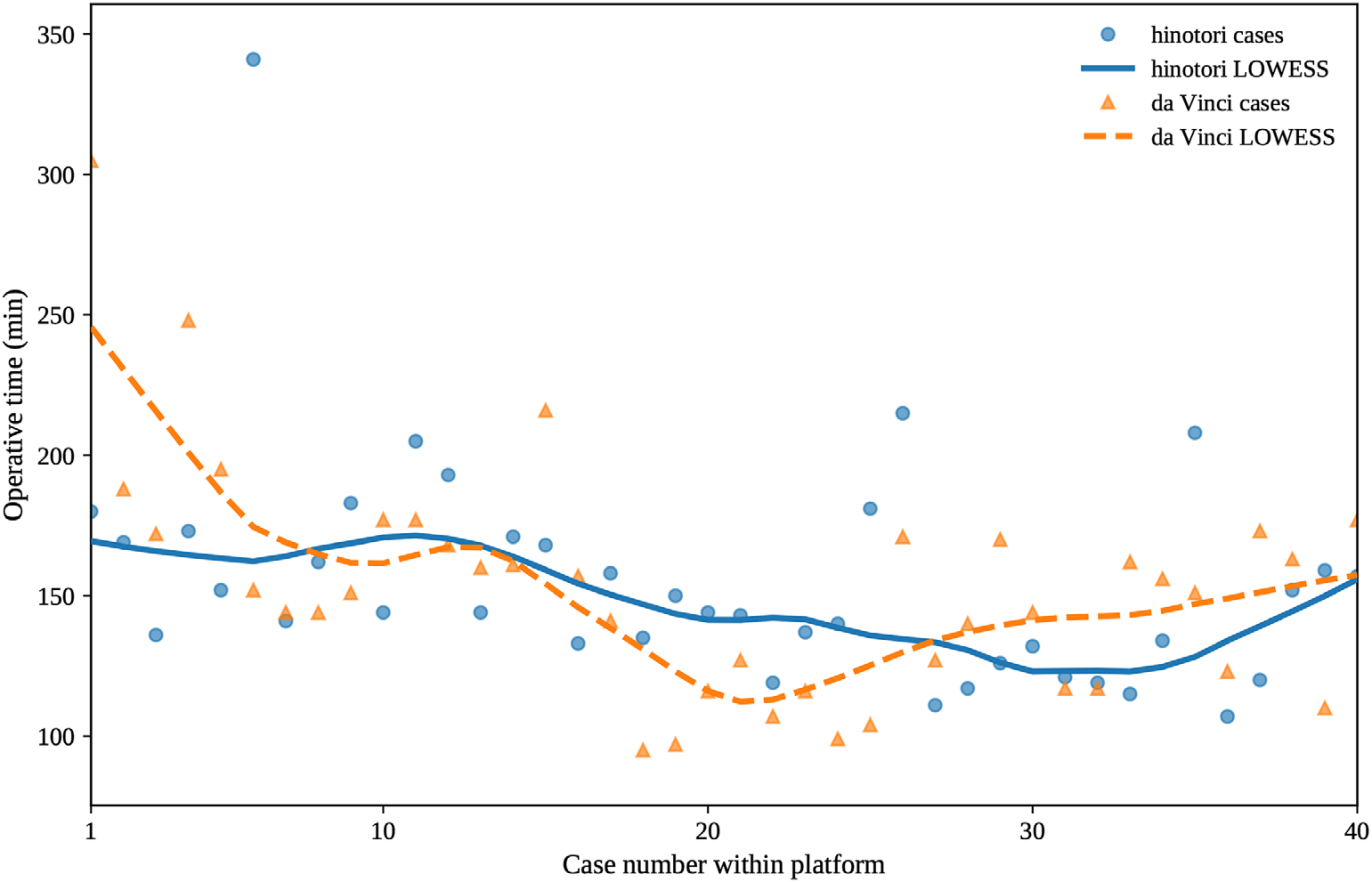
Learning curves for operative time during the early introduction phase. Operative time was plotted by sequential case number for the first 40 cases of each platform, with locally weighted scatterplot smoothing (LOWESS) curves overlaid to show temporal trends. Blue circles and a blue solid line represent the hinotori group, whereas orange triangles and an orange dashed line represent the da Vinci group.

Operative time was plotted according to the sequential case number within each platform to evaluate temporal trends during platform introduction. In the early 40‐case comparison, operative time showed a gradual downward trend in both platforms, but the hinotori group remained at a higher level than the da Vinci group throughout most of the introductory phase (Figure 3). In the full‐cohort supplementary analysis, operative time showed a progressive reduction over the longer institutional experience in the da Vinci cohort, whereas the hinotori cohort was limited to the introductory 40 cases (Figure S1).

## 4 Discussion

This study used PSM to compare the surgical outcomes in RSH for benign uterine diseases between the Japanese‐made hinotori surgical robot system and the preceding da Vinci system. The two groups showed no significant difference in the incidence of CD Grade I–V complications within 30 days postoperatively, which was the primary endpoint, and no CD Grade IIIa–V complications were observed in either group. However, operative and console times were significantly longer in the hinotori group. The persistence of longer operative and console times after PSM suggests that these time‐related differences were not fully explained by measurable preoperative baseline differences alone. These results suggest that RSH using the hinotori system for benign uterine diseases can be performed without apparent short‐term safety concerns in this matched cohort, although the longer operative and console times require further consideration.

The primary finding of this study is that the hinotori system showed no apparent short‐term safety concerns compared with the da Vinci system, which has an established record of success in gynecological surgery. Previous studies have compared surgical outcomes between the hinotori and da Vinci systems in gastrointestinal surgery and urology using PSM to adjust for background factors [11] [21]. The present study extends this comparative framework to RSH for benign uterine diseases, using PSM to adjust for observable differences in preoperative patient characteristics. PSM analyses in rectal cancer surgery have shown that the hinotori system provides outcomes comparable to those of the da Vinci system regarding serious complications, such as suture failure and organ damage, although the hinotori system tends to require longer operative times [22, 23]. Similarly, in robot‐assisted sacrocolpopexy, which involves procedures in the deep pelvis, the hinotori system has been found comparable to the da Vinci system and clinically acceptable in terms of safety, although operative time was longer with the hinotori system [24]. Furthermore, Kumamoto et al. reported that the docking‐free design and optimal trocar placement of the hinotori system may significantly reduce the risk of intraoperative subcutaneous emphysema compared with the da Vinci system, thereby enhancing patient safety [25].

Recent studies in gynecology have incorporated Medtronic's Hugo robotic‐assisted surgery system alongside the hinotori and da Vinci systems, enabling comparative analyses among these three models. Nagata et al. reported no significant differences among the three systems regarding perioperative complications or postoperative recovery in robot‐assisted total hysterectomy [18]. Similarly, Matsuura et al. found that early surgical outcomes were comparable across the three models [17]. Taken together with these previous reports, our findings add to the early clinical evidence supporting the short‐term safety and feasibility of hinotori‐assisted hysterectomy.

On postoperative Day 1, the CRP level was significantly higher in the hinotori group than in the da Vinci group. A recent study found that operative time and patient BMI are significant independent factors affecting postoperative CRP levels in laparoscopic hysterectomy and RSH [26]. Therefore, the elevated CRP levels in the hinotori group likely reflect a temporary response to surgical stress associated with the longer operative time, rather than excessive tissue damage caused by the hinotori procedure. In fact, no differences in clinically significant complications or postoperative hospital stay were observed between the two groups. This suggests that transient increases in CRP do not adversely affect patient prognosis or safety.

Certain technical features of the hinotori system may have supported the safe performance of RSH in this cohort. The system's eight‐axis arm configuration and compact arm structure help prevent arm‐to‐arm interference and are particularly beneficial for procedures in areas with a limited field of view, such as deep parts of the pelvis [9, 14, 15]. In addition, the docking‐free design has been suggested to potentially reduce postoperative port‐site pain and lower the incidence of subcutaneous emphysema by providing sufficient working space around the trocar and preventing the abdominal wall from being exposed to excessive traction [25]. Even when using a four‐port technique without an assistant port, as in this study, these arm operation characteristics may have supported the safe execution of surgery. In terms of ergonomics, console design affects the surgeon's burden, as noted by Krauss et al. [27]. The Surgeon Cockpit is ergonomically optimized with a 16:9 wide monitor that provides a large field of view and a 3D visualization system with flexible position adjustment, which may help reduce surgeon fatigue during long, complex surgeries [28, 29]. These potential advantages were not directly evaluated in the present study but may be relevant when considering the clinical utility of the hinotori platform beyond short‐term perioperative outcomes.

Although the hinotori system was associated with prolonged operative and console times, these delays did not appear to compromise patient safety in this cohort. Several workflow‐related factors may have contributed to the longer procedure‐related times. Due to its docking‐free design, the hinotori system necessitates manual pivot setting and placement adjustment [30]. However, the setup time also includes the process of setting the arms to safe positions optimized for each patient, which may contribute to reducing complications [25]. Setup‐related time may decrease as the surgical team gains proficiency [23]. Additionally, in the four‐port technique without an assistant port used in this study, needle insertion, needle retrieval, suture cutting, and vaginal cuff closure may require instrument replacement or additional team coordination. These workflow factors may have contributed to the longer operative and console times, particularly during the early phase of platform adoption. Therefore, the longer procedure‐related times should be interpreted as potentially reflecting platform‐specific workflow, the reduced‐port surgical setting, and early adoption effects, rather than as evidence of compromised patient safety.

The persistence of longer operative and console times after PSM suggests that these time‐related differences were not fully explained by measurable preoperative baseline differences alone. Because surgeon experience, institutional workflow, and early adoption effects were not fully captured by the propensity score model, the longer procedure‐related times should be interpreted together with the observed temporal trends in operative time. In the hinotori group, operative time tended to decrease over the first 40 cases, although the cohort still represented the introductory phase. Matsushita et al. compared surgical robotic systems in radical prostatectomy and reported that even surgeons experienced with the da Vinci system required longer operative times when transitioning to the hinotori system [12]. This finding suggests that surgeons may require an adaptation period to become accustomed to the unique operational characteristics and arm range of motion of the new system. Taken together, the longer operative and console times observed in the hinotori group may be partly related to the early stage of platform implementation.

The hinotori system offers scalability as a digital platform. Its AI‐supported analysis system enables anatomical structure recognition [31], and its successful application in telesurgery during distal gastrectomy is expected to standardize surgical procedures and enhance safety [28]. A supportive environment exists for the smooth introduction of these technologies in clinical settings, as surgeons familiar with existing robotic platforms can transition to the hinotori system relatively easily [12].

This study has four limitations. First, as a single‐center, retrospective observational study, it may have been subject to unknown confounding factors. Second, the analysis included only 40 cases per group, thereby reducing statistical power to detect rare complications. Third, the inclusion of only the first 40 hinotori cases after introduction limited our ability to separate the effects of platform‐specific workflow, surgeon adaptation, and institutional experience on operative time. Fourth, the post‐matching statistical tests did not explicitly account for the matched‐pair structure; therefore, *p*‐values in the matched cohort should be interpreted descriptively.

In this study using PSM analysis, RSH using the hinotori system showed no apparent short‐term safety concerns compared with the da Vinci system in patients with benign uterine diseases. Although operative and console times were longer in the hinotori group, these findings may be partly related to the early stage following its introduction and the adaptation required for the new robotic platform. The Japanese‐made hinotori system may become a useful option for robot‐assisted surgery in gynecology as clinical experience accumulates.

## Funding

The authors have nothing to report.

## Ethics Statement

The Medical Research Ethics Review Committee of the School of Medicine at Fujita Health University (Approval No. HM23‐251) approved this comprehensive research study, which was conducted in accordance with the principles of the Declaration of Helsinki. Participants were allowed to indicate their willingness not to consent to the study in an opt‐out format, and those who did not explicitly opt out were considered to have consented to use of their data in this study.

## Conflicts of Interest

The authors declare no conflicts of interest.

## Supporting information


**Figure S1:** Full‐cohort learning curves for operative time and console time.

## Data Availability

The data that support the findings of this study are available on request from the corresponding author. The data are not publicly available due to privacy or ethical restrictions.
