# Supplementary figures and images for: A Comparative Analysis of the Hinotori and da Vinci Robotic Systems in Total Hysterectomy for Benign Uterine Gynecological Diseases Using Propensity Score Matching
Source: Asian J Endosc Surg. 2026 Jun 14;19(1):e70327. doi: 10.1111/ases.70327 (PMC13265626; doi:10.1111/ases.70327)

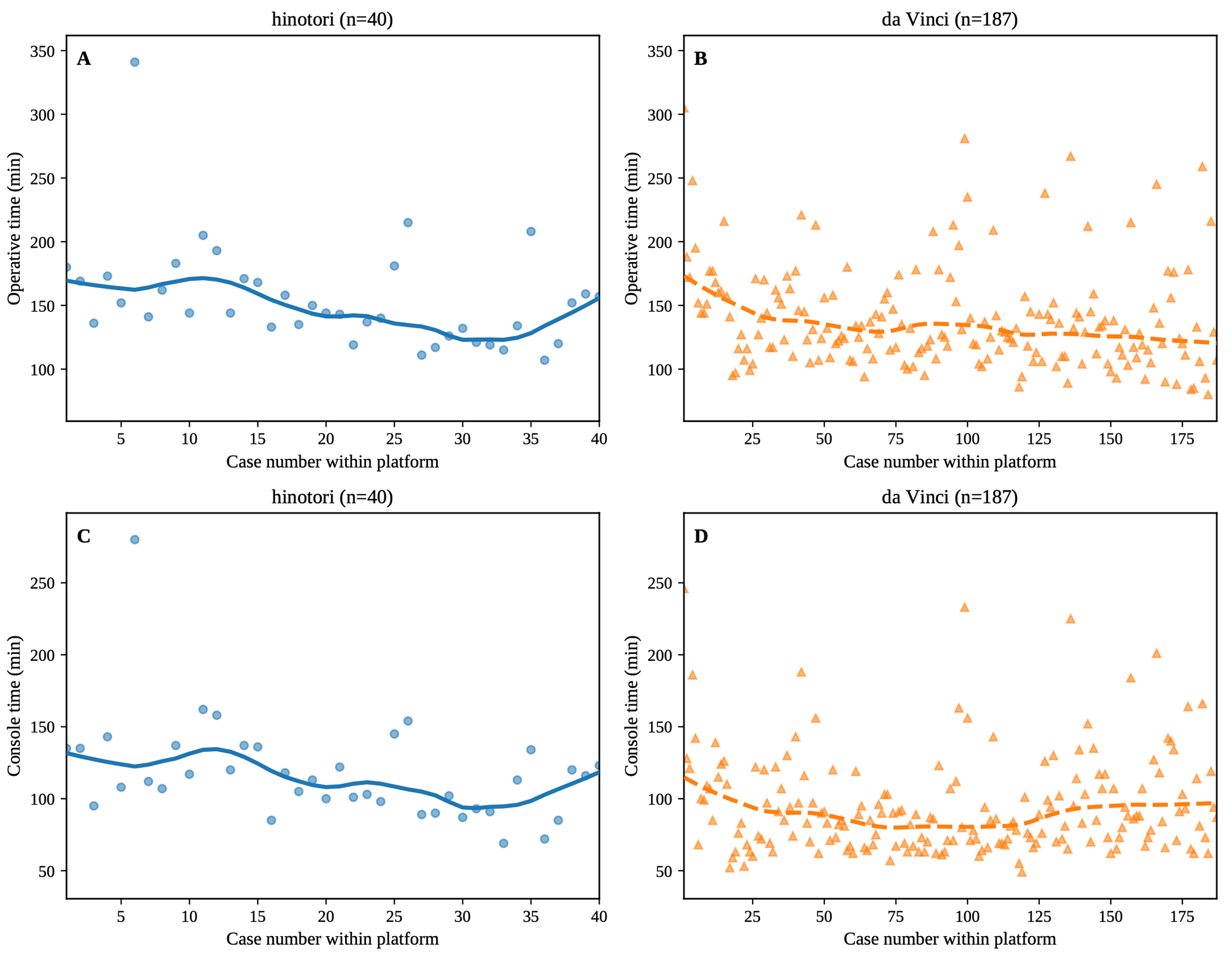

Supplement: Supplementary file 1 — Figure S1: Full‐cohort learning curves for operative time and console time. [file ASES-19-e70327-s001.tif]
